# Supplementary material for: Hot spot prediction in protein-protein interactions by an ensemble system
Source: BMC Syst Biol. 2018 Dec 31;12(Suppl 9):132. doi: 10.1186/s12918-018-0665-8 (PMC6311905; doi:10.1186/s12918-018-0665-8)
Supplement: Supplementary file 2 — All relevant MATLAB code. A simple Matlab implement of our predictor. (ZIP 19,742 kb) [file 12918_2018_665_MOESM2_ESM.zip › Additional file 2/Read ASA/ASA website/dbMPIKT/4PWXC.html]

 NetSurfP - Protein Surface Accessibility and Secondary Structure Predictions

  

|  |  |  |
| --- | --- | --- |
|  |  | NetSurfP - Protein Surface Accessibility and Secondary Structure Predictions  Technical University of Denmark |

  


---

```
# For publication of results, please cite:
# A generic method for assignment of reliability scores applied to solvent accessibility predictions.
# Bent Petersen, Thomas Nordahl Petersen, Pernille Andersen, Morten Nielsen and Claus Lundegaard
# BMC Structural Biology 2009, 9:51 doi:10.1186/1472-6807-9-51
#
# Column 1: Class assignment - B for buried or E for Exposed - Threshold: 25% exposure, but not based on RSA
# Column 2: Amino acid
# Column 3: Sequence name
# Column 4: Amino acid number
# Column 5: Relative Surface Accessibility - RSA
# Column 6: Absolute Surface Accessibility
# Column 7: Z-fit score for RSA prediction
# Column 8: Probability for Alpha-Helix
# Column 9: Probability for Beta-strand
# Column 10: Probability for Coil
E M  4PWXC                  1    0.788 157.739  -1.880   0.003   0.003   0.994
E G  4PWXC                  2    0.617  48.582  -1.850   0.109   0.005   0.886
E A  4PWXC                  3    0.612  67.464  -1.283   0.600   0.003   0.397
E K  4PWXC                  4    0.480  98.818   0.395   0.858   0.002   0.139
B L  4PWXC                  5    0.118  21.606  -0.690   0.858   0.002   0.139
E A  4PWXC                  6    0.440  48.455   0.835   0.879   0.010   0.111
E K  4PWXC                  7    0.510 104.845   1.474   0.938   0.007   0.055
B T  4PWXC                  8    0.092  12.774   0.640   0.970   0.001   0.030
B L  4PWXC                  9    0.141  25.744   0.756   0.988   0.000   0.012
E Q  4PWXC                 10    0.552  98.641   0.651   0.988   0.000   0.012
E R  4PWXC                 11    0.365  83.585   1.936   0.970   0.001   0.030
B F  4PWXC                 12    0.040   8.048   0.412   0.970   0.001   0.030
E E  4PWXC                 13    0.459  80.240   2.005   0.970   0.001   0.030
E N  4PWXC                 14    0.615  90.065   1.354   0.923   0.002   0.076
E K  4PWXC                 15    0.283  58.275   1.320   0.923   0.002   0.076
B I  4PWXC                 16    0.109  20.073  -0.047   0.782   0.003   0.216
E K  4PWXC                 17    0.676 138.991   0.656   0.600   0.003   0.397
E A  4PWXC                 18    0.734  80.887   0.750   0.246   0.004   0.750
E G  4PWXC                 19    0.497  39.130  -0.734   0.109   0.005   0.886
E D  4PWXC                 20    0.441  63.534   0.285   0.109   0.005   0.886
B Y  4PWXC                 21    0.224  47.933  -0.050   0.622   0.015   0.363
B Y  4PWXC                 22    0.191  40.838   0.499   0.879   0.010   0.111
E E  4PWXC                 23    0.330  57.564   0.145   0.938   0.007   0.055
B A  4PWXC                 24    0.057   6.314   0.144   0.970   0.001   0.030
B H  4PWXC                 25    0.089  16.225   0.288   0.970   0.001   0.030
B Q  4PWXC                 26    0.124  22.164  -0.205   0.970   0.001   0.030
B T  4PWXC                 27    0.181  25.063   0.381   0.970   0.001   0.030
B L  4PWXC                 28    0.042   7.617   0.553   0.988   0.000   0.012
B R  4PWXC                 29    0.122  27.961   0.741   0.988   0.000   0.012
B T  4PWXC                 30    0.072   9.945  -0.099   0.988   0.000   0.012
B I  4PWXC                 31    0.085  15.725   0.531   0.988   0.000   0.012
B A  4PWXC                 32    0.066   7.284  -0.502   0.988   0.000   0.012
B N  4PWXC                 33    0.145  21.272  -0.044   0.970   0.001   0.030
B R  4PWXC                 34    0.240  54.937   0.383   0.970   0.001   0.030
B Y  4PWXC                 35    0.166  35.496  -0.247   0.923   0.002   0.076
B V  4PWXC                 36    0.173  26.529   0.274   0.879   0.010   0.111
E R  4PWXC                 37    0.490 112.164   0.803   0.600   0.003   0.397
E S  4PWXC                 38    0.517  60.639   0.545   0.321   0.003   0.675
E K  4PWXC                 39    0.648 133.376   0.415   0.109   0.005   0.886
E S  4PWXC                 40    0.483  56.584   1.021   0.016   0.005   0.979
B Y  4PWXC                 41    0.154  32.910   0.992   0.600   0.003   0.397
E E  4PWXC                 42    0.700 122.290   1.361   0.923   0.002   0.076
E H  4PWXC                 43    0.391  71.050   1.089   0.970   0.001   0.030
B A  4PWXC                 44    0.018   1.973   0.751   0.970   0.001   0.030
B I  4PWXC                 45    0.100  18.518   0.907   0.988   0.000   0.012
E E  4PWXC                 46    0.451  78.790   1.384   0.988   0.000   0.012
B L  4PWXC                 47    0.072  13.147   1.238   0.988   0.000   0.012
B I  4PWXC                 48    0.027   5.013   0.675   0.988   0.000   0.012
B S  4PWXC                 49    0.165  19.361   0.153   0.988   0.000   0.012
E Q  4PWXC                 50    0.389  69.404   0.384   0.970   0.001   0.030
B G  4PWXC                 51    0.061   4.777  -0.040   0.970   0.001   0.030
B A  4PWXC                 52    0.018   1.973   0.986   0.970   0.001   0.030
E L  4PWXC                 53    0.244  44.603   0.926   0.970   0.001   0.030
E S  4PWXC                 54    0.273  32.019   1.257   0.970   0.001   0.030
B F  4PWXC                 55    0.058  11.560   0.596   0.970   0.001   0.030
B L  4PWXC                 56    0.087  16.003   0.317   0.923   0.002   0.076
E K  4PWXC                 57    0.624 128.398   1.562   0.858   0.002   0.139
E A  4PWXC                 58    0.372  41.016   0.088   0.502   0.002   0.495
E K  4PWXC                 59    0.497 102.171  -0.755   0.176   0.004   0.820
E Q  4PWXC                 60    0.279  49.812  -0.801   0.176   0.004   0.820
E G  4PWXC                 61    0.305  24.035  -1.127   0.339   0.016   0.645
B G  4PWXC                 62    0.120   9.444  -1.728   0.455   0.046   0.498
B S  4PWXC                 63    0.048   5.579  -0.887   0.660   0.049   0.291
B G  4PWXC                 64    0.024   1.889  -0.857   0.938   0.007   0.055
B T  4PWXC                 65    0.038   5.340  -0.667   0.970   0.001   0.030
B D  4PWXC                 66    0.087  12.479  -0.322   0.970   0.001   0.030
B L  4PWXC                 67    0.022   3.937   1.160   0.988   0.000   0.012
B I  4PWXC                 68    0.018   3.386   0.777   0.988   0.000   0.012
B F  4PWXC                 69    0.103  20.672   0.610   0.988   0.000   0.012
B Y  4PWXC                 70    0.088  18.720   0.595   0.988   0.000   0.012
B L  4PWXC                 71    0.026   4.724   0.823   0.975   0.003   0.022
B L  4PWXC                 72    0.037   6.683   0.448   0.975   0.003   0.022
E E  4PWXC                 73    0.309  54.070   0.667   0.975   0.003   0.022
B V  4PWXC                 74    0.047   7.270   0.771   0.975   0.003   0.022
B Y  4PWXC                 75    0.093  19.895   0.448   0.938   0.007   0.055
E D  4PWXC                 76    0.533  76.805   1.148   0.879   0.010   0.111
E L  4PWXC                 77    0.604 110.574   1.295   0.717   0.014   0.269
E A  4PWXC                 78    0.427  47.099  -0.560   0.176   0.004   0.820
E E  4PWXC                 79    0.746 130.414   0.182   0.018   0.019   0.964
B V  4PWXC                 80    0.201  30.971   1.250   0.018   0.047   0.935
E K  4PWXC                 81    0.594 122.227   0.982   0.019   0.141   0.840
B V  4PWXC                 82    0.109  16.738   0.416   0.018   0.088   0.893
E D  4PWXC                 83    0.399  57.496   0.322   0.005   0.045   0.951
E D  4PWXC                 84    0.672  96.850  -0.276   0.782   0.003   0.216
E I  4PWXC                 85    0.681 125.911   1.085   0.858   0.002   0.139
B S  4PWXC                 86    0.103  12.118   0.253   0.858   0.002   0.139
B V  4PWXC                 87    0.109  16.799   0.815   0.970   0.001   0.030
E A  4PWXC                 88    0.582  64.114   0.889   0.970   0.001   0.030
E R  4PWXC                 89    0.334  76.600   1.845   0.988   0.000   0.012
B L  4PWXC                 90    0.016   2.893   1.322   0.988   0.000   0.012
B V  4PWXC                 91    0.153  23.516   0.728   0.988   0.000   0.012
E R  4PWXC                 92    0.476 109.119   1.590   0.988   0.000   0.012
B L  4PWXC                 93    0.037   6.848   0.231   0.970   0.001   0.030
B I  4PWXC                 94    0.064  11.895   0.304   0.938   0.007   0.055
E A  4PWXC                 95    0.472  51.992   1.239   0.879   0.010   0.111
E E  4PWXC                 96    0.431  75.313   1.232   0.600   0.003   0.397
B L  4PWXC                 97    0.041   7.599   0.567   0.176   0.004   0.820
E D  4PWXC                 98    0.437  62.914   0.777   0.016   0.005   0.979
E P  4PWXC                 99    0.737 104.609  -0.418   0.003   0.003   0.994
E S  4PWXC                100    0.694  81.278  -0.870   0.016   0.005   0.979
E E  4PWXC                101    0.433  75.698  -0.287   0.053   0.005   0.942
E P  4PWXC                102    0.490  69.545  -0.723   0.406   0.004   0.590
E N  4PWXC                103    0.495  72.497   0.320   0.600   0.003   0.397
B L  4PWXC                104    0.151  27.703   0.488   0.923   0.002   0.076
E K  4PWXC                105    0.317  65.289   1.541   0.970   0.001   0.030
E D  4PWXC                106    0.487  70.220   2.088   0.970   0.001   0.030
B V  4PWXC                107    0.051   7.808   1.136   0.988   0.000   0.012
B I  4PWXC                108    0.018   3.367   1.410   0.988   0.000   0.012
E T  4PWXC                109    0.449  62.207   1.717   0.988   0.000   0.012
E G  4PWXC                110    0.315  24.775   1.578   0.970   0.001   0.030
B M  4PWXC                111    0.024   4.822   0.370   0.970   0.001   0.030
B N  4PWXC                112    0.099  14.552   0.531   0.970   0.001   0.030
E N  4PWXC                113    0.468  68.574   1.396   0.923   0.002   0.076
B W  4PWXC                114    0.097  23.353   0.621   0.858   0.002   0.139
B S  4PWXC                115    0.039   4.536  -1.519   0.782   0.003   0.216
E I  4PWXC                116    0.423  78.163  -0.091   0.694   0.003   0.303
E K  4PWXC                117    0.614 126.362   0.390   0.622   0.015   0.363
E F  4PWXC                118    0.311  62.498  -0.462   0.246   0.004   0.750
E S  4PWXC                119    0.502  58.811  -2.178   0.053   0.005   0.942
E E  4PWXC                120    0.770 134.484  -0.147   0.016   0.005   0.979
E Y  4PWXC                121    0.292  62.465   0.164   0.018   0.019   0.964
E K  4PWXC                122    0.567 116.652  -1.039   0.018   0.019   0.964
E F  4PWXC                123    0.425  85.237  -0.373   0.018   0.047   0.935
B G  4PWXC                124    0.205  16.149  -1.778   0.018   0.019   0.964
B D  4PWXC                125    0.200  28.777   0.954   0.053   0.005   0.942
E P  4PWXC                126    0.512  72.624  -0.570   0.923   0.002   0.076
E Y  4PWXC                127    0.347  74.154   1.082   0.970   0.001   0.030
B L  4PWXC                128    0.022   4.101   1.256   0.970   0.001   0.030
B H  4PWXC                129    0.058  10.532   1.046   0.970   0.001   0.030
E N  4PWXC                130    0.363  53.187   1.204   0.988   0.000   0.012
B T  4PWXC                131    0.118  16.422   1.263   0.988   0.000   0.012
B I  4PWXC                132    0.024   4.403   1.145   0.988   0.000   0.012
B G  4PWXC                133    0.033   2.581  -0.417   0.988   0.000   0.012
E S  4PWXC                134    0.254  29.816   1.235   0.970   0.001   0.030
B K  4PWXC                135    0.156  32.171   1.866   0.970   0.001   0.030
B L  4PWXC                136    0.058  10.620   1.055   0.975   0.003   0.022
B L  4PWXC                137    0.119  21.807   0.261   0.938   0.007   0.055
E E  4PWXC                138    0.554  96.766   1.491   0.717   0.014   0.269
E G  4PWXC                139    0.618  48.597   0.015   0.181   0.016   0.803
E D  4PWXC                140    0.563  81.114   0.102   0.016   0.005   0.979
E F  4PWXC                141    0.423  84.836   0.121   0.115   0.016   0.868
B V  4PWXC                142    0.204  31.324  -0.136   0.605   0.105   0.290
B Y  4PWXC                143    0.276  58.938  -0.396   0.701   0.107   0.192
E E  4PWXC                144    0.239  41.823   0.675   0.701   0.107   0.192
B A  4PWXC                145    0.027   3.019  -0.287   0.701   0.107   0.192
B E  4PWXC                146    0.175  30.555   0.096   0.725   0.163   0.112
E R  4PWXC                147    0.332  76.097   0.621   0.701   0.107   0.192
B Y  4PWXC                148    0.082  17.438   0.731   0.605   0.105   0.290
B F  4PWXC                149    0.043   8.570   0.775   0.578   0.229   0.194
B M  4PWXC                150    0.050   9.945  -0.171   0.478   0.309   0.214
B L  4PWXC                151    0.144  26.348   0.454   0.352   0.332   0.316
B G  4PWXC                152    0.107   8.429  -0.631   0.125   0.227   0.648
B T  4PWXC                153    0.244  33.871   0.179   0.052   0.084   0.864
E H  4PWXC                154    0.486  88.476  -0.845   0.053   0.043   0.903
E D  4PWXC                155    0.526  75.854  -1.941   0.115   0.016   0.868
E S  4PWXC                156    0.336  39.426  -1.167   0.109   0.005   0.886
B M  4PWXC                157    0.070  13.927  -1.291   0.923   0.002   0.076
E I  4PWXC                158    0.371  68.653   0.106   0.970   0.001   0.030
B K  4PWXC                159    0.222  45.665   0.680   0.970   0.001   0.030
B Y  4PWXC                160    0.047  10.151   0.183   0.970   0.001   0.030
B V  4PWXC                161    0.029   4.488   0.240   0.988   0.000   0.012
E D  4PWXC                162    0.377  54.254   1.393   0.988   0.000   0.012
B L  4PWXC                163    0.072  13.110   0.820   0.988   0.000   0.012
B L  4PWXC                164    0.023   4.211   1.063   0.988   0.000   0.012
B W  4PWXC                165    0.131  31.602   1.060   0.988   0.000   0.012
E D  4PWXC                166    0.409  58.980   1.376   0.988   0.000   0.012
B W  4PWXC                167    0.059  14.069   0.668   0.970   0.001   0.030
B L  4PWXC                168    0.152  27.776  -0.050   0.923   0.002   0.076
E C  4PWXC                169    0.456  64.036   0.607   0.858   0.002   0.139
E Q  4PWXC                170    0.460  82.210   0.558   0.717   0.014   0.269
B V  4PWXC                171    0.119  18.275  -0.710   0.430   0.016   0.555
E D  4PWXC                172    0.457  65.897  -0.155   0.257   0.016   0.727
E D  4PWXC                173    0.689  99.242  -0.700   0.115   0.016   0.868
E I  4PWXC                174    0.647 119.787  -2.138   0.053   0.005   0.942
E E  4PWXC                175    0.388  67.853  -0.970   0.058   0.017   0.925
E D  4PWXC                176    0.520  74.961   0.078   0.058   0.017   0.925
E S  4PWXC                177    0.283  33.191  -0.871   0.181   0.016   0.803
B T  4PWXC                178    0.283  39.321  -0.702   0.339   0.016   0.645
B V  4PWXC                179    0.123  18.967   0.238   0.782   0.003   0.216
B A  4PWXC                180    0.082   9.069  -1.119   0.858   0.002   0.139
B E  4PWXC                181    0.073  12.806   0.363   0.938   0.007   0.055
B F  4PWXC                182    0.037   7.366  -0.236   0.975   0.003   0.022
B F  4PWXC                183    0.029   5.901   0.212   0.970   0.001   0.030
B S  4PWXC                184    0.022   2.602   0.578   0.970   0.001   0.030
B R  4PWXC                185    0.094  21.618   0.294   0.970   0.001   0.030
B L  4PWXC                186    0.043   7.928  -0.267   0.970   0.001   0.030
B V  4PWXC                187    0.030   4.626   0.517   0.970   0.001   0.030
B F  4PWXC                188    0.029   5.760   0.724   0.970   0.001   0.030
B N  4PWXC                189    0.083  12.107   0.504   0.970   0.001   0.030
B Y  4PWXC                190    0.061  13.014   0.326   0.923   0.002   0.076
B L  4PWXC                191    0.039   7.178  -0.198   0.923   0.002   0.076
B F  4PWXC                192    0.086  17.361  -0.609   0.782   0.003   0.216
B I  4PWXC                193    0.155  28.749  -0.807   0.406   0.004   0.590
E S  4PWXC                194    0.442  51.838  -0.677   0.176   0.004   0.820
E N  4PWXC                195    0.319  46.687  -0.237   0.053   0.005   0.942
B I  4PWXC                196    0.185  34.151   0.577   0.717   0.014   0.269
E S  4PWXC                197    0.557  65.327   0.518   0.879   0.010   0.111
B F  4PWXC                198    0.219  43.913   0.625   0.923   0.002   0.076
B A  4PWXC                199    0.013   1.488   0.917   0.970   0.001   0.030
E H  4PWXC                200    0.288  52.424   0.905   0.988   0.000   0.012
E E  4PWXC                201    0.412  72.064   1.529   0.988   0.000   0.012
B S  4PWXC                202    0.038   4.418  -0.372   0.988   0.000   0.012
B K  4PWXC                203    0.054  11.211   0.817   0.988   0.000   0.012
E D  4PWXC                204    0.412  59.441   1.520   0.988   0.000   0.012
E I  4PWXC                205    0.290  53.576   1.785   0.988   0.000   0.012
B F  4PWXC                206    0.041   8.209   0.146   0.970   0.001   0.030
B L  4PWXC                207    0.092  16.790   0.295   0.970   0.001   0.030
E E  4PWXC                208    0.519  90.652   1.196   0.923   0.002   0.076
E R  4PWXC                209    0.398  91.211   1.099   0.858   0.002   0.139
B F  4PWXC                210    0.056  11.219   0.393   0.782   0.003   0.216
B I  4PWXC                211    0.250  46.213  -0.288   0.600   0.003   0.397
E E  4PWXC                212    0.699 122.045  -0.185   0.600   0.003   0.397
E K  4PWXC                213    0.580 119.285  -0.479   0.430   0.016   0.555
E F  4PWXC                214    0.367  73.617  -0.959   0.257   0.016   0.727
E H  4PWXC                215    0.415  75.507  -0.845   0.058   0.017   0.925
E P  4PWXC                216    0.411  58.378  -1.239   0.018   0.019   0.964
E K  4PWXC                217    0.602 123.852  -0.123   0.052   0.084   0.864
B Y  4PWXC                218    0.195  41.736  -0.337   0.064   0.216   0.721
E E  4PWXC                219    0.446  77.864  -0.971   0.066   0.296   0.638
E K  4PWXC                220    0.537 110.481  -0.413   0.064   0.216   0.721
E I  4PWXC                221    0.356  65.768  -1.421   0.056   0.142   0.802
E D  4PWXC                222    0.514  74.082  -0.976   0.052   0.084   0.864
E K  4PWXC                223    0.603 124.078  -1.626   0.018   0.047   0.935
E N  4PWXC                224    0.552  80.769  -3.036   0.018   0.019   0.964
E G  4PWXC                225    0.584  45.977  -2.547   0.018   0.019   0.964
E Y  4PWXC                226    0.388  82.873  -1.552   0.018   0.019   0.964
E E  4PWXC                227    0.558  97.448  -1.534   0.018   0.088   0.893
B I  4PWXC                228    0.205  37.907  -1.148   0.021   0.279   0.699
E V  4PWXC                229    0.364  55.901  -0.756   0.021   0.279   0.699
E F  4PWXC                230    0.332  66.673  -1.172   0.021   0.279   0.699
B F  4PWXC                231    0.180  36.046  -0.872   0.019   0.141   0.840
E E  4PWXC                232    0.404  70.561  -1.025   0.018   0.047   0.935
E D  4PWXC                233    0.458  66.027  -1.766   0.018   0.019   0.964
B Y  4PWXC                234    0.190  40.518   0.277   0.176   0.004   0.820
B S  4PWXC                235    0.162  18.986  -0.448   0.406   0.004   0.590
B D  4PWXC                236    0.065   9.309  -0.843   0.782   0.003   0.216
B L  4PWXC                237    0.036   6.537  -0.106   0.858   0.002   0.139
B N  4PWXC                238    0.068   9.999  -0.377   0.923   0.002   0.076
B F  4PWXC                239    0.033   6.583  -0.092   0.970   0.001   0.030
B L  4PWXC                240    0.037   6.738  -0.298   0.970   0.001   0.030
B Q  4PWXC                241    0.155  27.612   0.176   0.988   0.000   0.012
B L  4PWXC                242    0.043   7.928   0.263   0.988   0.000   0.012
B L  4PWXC                243    0.033   6.006   0.480   0.988   0.000   0.012
B L  4PWXC                244    0.049   8.990   0.855   0.988   0.000   0.012
B I  4PWXC                245    0.112  20.683   1.112   0.970   0.001   0.030
B T  4PWXC                246    0.054   7.545   0.655   0.975   0.003   0.022
B C  4PWXC                247    0.038   5.335   0.647   0.938   0.007   0.055
E Q  4PWXC                248    0.335  59.867   1.035   0.717   0.014   0.269
E T  4PWXC                249    0.476  66.035   0.640   0.246   0.004   0.750
E A  4PWXC                250    0.678  74.760   0.380   0.053   0.005   0.942
E D  4PWXC                251    0.672  96.850   0.825   0.016   0.005   0.979
B A  4PWXC                252    0.197  21.731   0.411   0.502   0.002   0.495
E S  4PWXC                253    0.670  78.559   0.591   0.782   0.003   0.216
E Y  4PWXC                254    0.243  52.015   1.530   0.858   0.002   0.139
B F  4PWXC                255    0.034   6.864  -0.271   0.970   0.001   0.030
E L  4PWXC                256    0.379  69.322   1.856   0.970   0.001   0.030
E N  4PWXC                257    0.450  65.851   1.805   0.988   0.000   0.012
B L  4PWXC                258    0.038   6.921   0.394   0.970   0.001   0.030
B K  4PWXC                259    0.120  24.705   1.370   0.988   0.000   0.012
E N  4PWXC                260    0.631  92.320   1.247   0.970   0.001   0.030
E H  4PWXC                261    0.365  66.375   2.103   0.970   0.001   0.030
B Y  4PWXC                262    0.046   9.916   0.539   0.923   0.002   0.076
B L  4PWXC                263    0.244  44.768   0.570   0.858   0.002   0.139
E D  4PWXC                264    0.723 104.170   0.940   0.782   0.003   0.216
E F  4PWXC                265    0.295  59.227   0.782   0.694   0.003   0.303
B S  4PWXC                266    0.135  15.775  -0.369   0.406   0.004   0.590
E Q  4PWXC                267    0.628 112.232   0.209   0.694   0.003   0.303
E A  4PWXC                268    0.451  49.733   1.243   0.782   0.003   0.216
B Y  4PWXC                269    0.050  10.728   0.988   0.923   0.002   0.076
E K  4PWXC                270    0.456  93.737   1.367   0.970   0.001   0.030
E S  4PWXC                271    0.573  67.156   1.702   0.970   0.001   0.030
B E  4PWXC                272    0.067  11.775   0.577   0.970   0.001   0.030
B L  4PWXC                273    0.039   7.068   0.598   0.970   0.001   0.030
E E  4PWXC                274    0.503  87.927   1.330   0.970   0.001   0.030
E F  4PWXC                275    0.393  78.895   1.586   0.970   0.001   0.030
B L  4PWXC                276    0.041   7.544   0.560   0.923   0.002   0.076
B G  4PWXC                277    0.128  10.097   0.089   0.923   0.002   0.076
E Q  4PWXC                278    0.492  87.853   0.620   0.879   0.010   0.111
E E  4PWXC                279    0.330  57.634   1.163   0.831   0.044   0.125
B Y  4PWXC                280    0.151  32.333   0.609   0.701   0.107   0.192
B F  4PWXC                281    0.178  35.765  -0.153   0.386   0.097   0.517
E N  4PWXC                282    0.646  94.589  -0.826   0.113   0.043   0.844
B E  4PWXC                283    0.136  23.777   0.278   0.053   0.043   0.903
E N  4PWXC                284    0.584  85.454   0.584   0.052   0.084   0.864
E L  4PWXC                285    0.315  57.640   1.073   0.053   0.043   0.903
E Y  4PWXC                286    0.475 101.465  -0.397   0.053   0.043   0.903
E F  4PWXC                287    0.614 123.170  -0.495   0.053   0.043   0.903
E Q  4PWXC                288    0.838 149.685   0.038   0.003   0.003   0.994

Explain the output.  Go back.
```

---
